# Sarcopenia during COVID-19 lockdown restrictions: long-term health effects of short-term muscle loss

**DOI:** 10.1007/s11357-020-00272-3

**Published:** 2020-10-01

**Authors:** Richard Kirwan, Deaglan McCullough, Tom Butler, Fatima Perez de Heredia, Ian G. Davies, Claire Stewart

**Affiliations:** 1 School of Biological and Environmental Sciences, Liverpool John Moores University, Liverpool, UK; 2 Research Institute of Sport and Exercise Science, Liverpool John Moores University, Liverpool, UK; 3 Department of Clinical Sciences and Nutrition, University of Chester, Chester, UK

**Keywords:** COVID-19, Sarcopenia, Obesity, Inflammation, Physical activity, Appetite regulation

## Abstract

The COVID-19 pandemic is an extraordinary global emergency that has led to the implementation of unprecedented measures in order to stem the spread of the infection. Internationally, governments are enforcing measures such as travel bans, quarantine, isolation, and social distancing leading to an extended period of time at home. This has resulted in reductions in physical activity and changes in dietary intakes that have the potential to accelerate sarcopenia, a deterioration of muscle mass and function (more likely in older populations), as well as increases in body fat. These changes in body composition are associated with a number of chronic, lifestyle diseases including cardiovascular disease (CVD), diabetes, osteoporosis, frailty, cognitive decline, and depression. Furthermore, CVD, diabetes, and elevated body fat are associated with greater risk of COVID-19 infection and more severe symptomology, underscoring the importance of avoiding the development of such morbidities. Here we review mechanisms of sarcopenia and their relation to the current data on the effects of COVID-19 confinement on physical activity, dietary habits, sleep, and stress as well as extended bed rest due to COVID-19 hospitalization. The potential of these factors to lead to an increased likelihood of muscle loss and chronic disease will be discussed. By offering a number of home-based strategies including resistance exercise, higher protein intakes and supplementation, we can potentially guide public health authorities to avoid a lifestyle disease and rehabilitation crisis post-COVID-19. Such strategies may also serve as useful preventative measures for reducing the likelihood of sarcopenia in general and in the event of future periods of isolation.

## Introduction

Sarcopenia is the age-associated decline in muscle mass, strength, and quality that begins as early as the fourth decade of life and is a major contributor to poor health and disability in older adults [1, 2]. The progressive loss of muscle mass and the concomitant decline in muscle strength (dynapenia) are associated with a large and diverse group of pathologies including type 2 diabetes mellitus (T2DM) [3], cardiovascular disease (CVD) [4], frailty and disability [5, 6], increased risk of falls and fractures [7, 8], loss of physical independence [9], cognitive decline and depression [10, 11], lower quality of life [12], and all-cause mortality [13, 14]. The etiology of this muscle loss is known to be multifactorial with reductions in activity levels and inappropriate nutrition playing central roles [15–20].

The COVID-19 pandemic is an extraordinary global emergency with over 26.5 million confirmed cases and more than 870,000 deaths as of September 5, 2020 [21], which has led to the implementation of unprecedented measures in order to stem the spread of the infection. Internationally, governments are recommending and/or enforcing such measures as travel bans, quarantine, isolation, and social distancing [22, 23] which in practice have resulted in an extended period of time spent in one’s place of residence. This has resulted in reductions in physical activity (PA) and increases in sedentary behavior [24, 25] which are associated with the loss of muscle mass [26]. Furthermore, hospitalization from COVID-19 can lead to extended bed rest with some recent reports noting average hospital stays of 11 days [27]. More severe presentation of COVID-19 infection can result in admission to intensive care units (ICUs) or requirement for invasive mechanical ventilation (IMV) [28, 29]. This can result in further restricted movement with reports of median length of ICU stay as 8 days with an interquartile range (IQR) up to 12 days [27]. Such extended periods of bed rest, as a result of COVID-19 isolation/quarantine or hospitalization, pose a further risk to muscle loss, particularly to older individuals [30]. This is of particular relevance given the higher rates of hospitalization reported in older individuals (≥ 65 years) [31].

Access to food has also been affected due to the pandemic with older populations and lower socio-economic groups in particular, experiencing the most relevant disruptions [32, 33]. Furthermore, quarantine and social isolation are known to result in increased levels of stress and anxiety [34–36] the consequence of which may be increased markers of atrophy and elevated loss of muscle mass [37]. This psychological stress may also lead to poorer dietary choices with a switch to hyperpalatable, convenience foods that are simultaneously high in sugar and/or fat [38] and which may displace more nutrient dense foods, reducing dietary protein intake [39]. Such dietary changes are also associated with poorer markers of cardiometabolic risk including overweight/obesity, hypertension, dyslipidemia, and other features of metabolic syndrome [40].

In this article, we will discuss how this combination of reduced physical activity and poorer diet quality, along with other lifestyle-related factors and the risk of hospitalization, has the potential to accelerate the loss of muscle and physical function. The long-lasting, deleterious effects of this muscle loss on multiple aspects of metabolic, physical, and psychological health will be discussed.

## Effects of COVID-19 restrictions, social distancing, and confinement on skeletal muscle mass

### Inactivity, sedentary behavior, and muscle loss

Prior to this pandemic, World Health Organization recommendations for PA (150 min/week of moderate-intensity aerobic PA with muscle strengthening exercises 2 day/week, etc.) were not being met, particularly in older populations [41]. COVID-19 presents a number of risks for further reductions in activity levels for the general population. Quarantine, self-isolation, social distancing, and other government measures have led to the closure of gyms and leisure centers as well as the suspension of group exercise and rehabilitation programs. It has never been easier to be physically inactive. In addition to and independently of reduced PA, increased sitting time and sedentary behavior, which have been reported to increase during COVID-19 confinement [25], are also associated with multiple adverse health outcomes [42], further compounding the risk to health.

Recently published research in children and adolescents (baseline age range 6–18 years) living through COVID-19 quarantine in Italy has shown a decrease in sports activity of 2.3 h and an increase in electronic device/screen time of 4.85 h per day. Similarly, a survey of 1047 participants from Asia, Africa, and Europe reported a 33.5% decrease in the number of minutes/day of PA, a decrease in metabolic equivalents of task (MET) values (a measure of exercise intensity) of 42.7%, and an increase in sitting time from 5 to 8 h per day [25]. The results of these studies highlight the potentially detrimental effect of quarantine/self-isolation on physical activity and sedentary behavior [43]. Hospitalization due to COVID-19, and in particular admission to ICU, can result in much lower levels of activity or even complete immobilization [27–29], which may greatly accelerate the loss of muscle mass and function in those affected [44]. Furthermore, some governmental recommendations on social distancing have advised particular stringency in older adults [45], who are deemed clinically vulnerable [46], meaning physical activity in this group may be even further reduced compared to the general population. Concerns around a “second-wave” of COVID-19 infections that is expected to follow a relaxation of current lockdown restrictions [47] may also result in such at-risk populations enduring significantly decreased physical activity for longer periods of time.

**Fig. 1:**
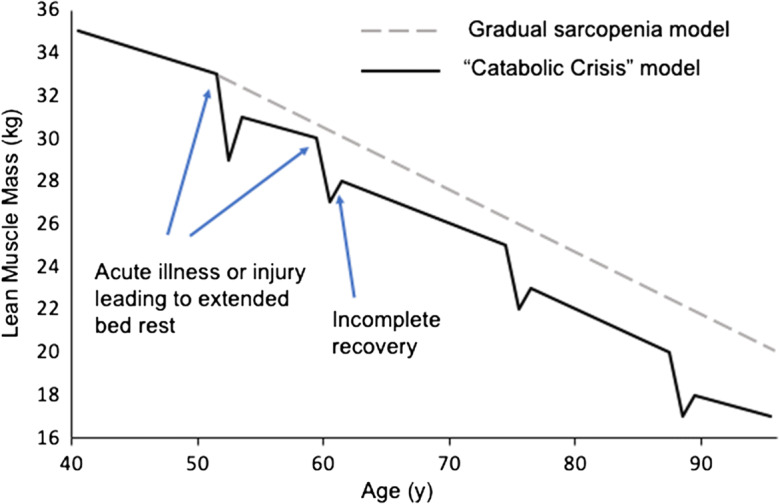
Potential model of age-associated muscle loss (sarcopenia) exacerbated by periods of extended bed rest/hospitalization due to acute illness or injury (catabolic crises). Adapted from English and Paddon-Jones (2010) [30]

Even short periods of reduced activity (both immobilization, simulating bed rest or hospitalization, and step reduction, which may better model COVID-19 confinement) have been shown to result in the rapid loss of muscle mass and physical function, even in younger adults [26, 48]. As much as 1.7% of muscle volume can be lost after as little as 2 days of immobilization, with greater losses (5.5% of muscle volume) observed after only 7 days [49]. A recent study using smartphone data from 1062 participants in 5 European countries observed that individuals had lower step counts and heart rates and spent more time in sedentary activity such as using their phones during COVID-19 lockdown [24]. The sudden reduction in activity and increase in sedentarism brought on by COVID-19 measures would closely mirror the “catabolic crisis” model of sarcopenia, proposed by English and Paddon-Jones [30]. In this model, sarcopenia is not simply a gradual process, but is in fact accelerated by periodic occasions of inactivity (such as periods of extended bed rest or hospitalization) (Fig. 1). Indeed, in a study of 118 ICU patients (mean age 55 years), muscle thickness measured by ultrasonography was negatively correlated with length of stay in ICU with loss of muscle thickness higher during the first 2–3 weeks of immobilization [50]. With ICU durations of up to 12 days being observed in some COVID-19-infected patients [27], the loss of muscle mass is a very likely scenario. The lean tissue lost during these times of inactivity may not be fully regained leading to a progressive loss of muscle mass and function. Highlighting this, in a study of 27 ICU patients (age range 23–78 years), both muscle mass and strength were decreased 7 days after ICU discharge and, while significantly improved after 6 months, did not normalize in the majority of patients [51].

The age-related decline in muscle mass is primarily due to the selective atrophy of type II fibers [52, 53]. This decline may be attributed to neurodegeneration of the skeletal muscle fiber, thereby reducing the potential to recruit type II fibers during resistance exercise (RE), resulting in a diminished anabolic response [54]. Indeed, regular RE has been shown to reduce this decline in type II fibers [53, 54]. Additionally, the rapid loss of muscle related to inactivity may be due to a number of further mechanisms including induced anabolic resistance, insulin resistance (IR), mitochondrial dysfunction, and its associated oxidative stress [44, 55–58]. Interestingly, this inactivity-induced reduction in skeletal muscle–associated muscle protein synthesis (MPS) can be rescued with RE and sufficient protein ingestion [59, 60] offering practical solutions to overcoming this driver of muscle loss (which will be discussed later in this article). Indeed, older individuals who have engaged in life-long RE/strength training have significantly greater rates of force development and increased muscle size compared with untrained control individuals [61]. This increased muscle size was predominantly attributed to type II muscle fibers, the loss of which is responsible for the decrease in muscle mass seen in sarcopenia [62].

Another potential, although indirect, mechanism by which reduced PA as a consequence of self-isolation may be detrimental to muscle mass is through the role of inactivity in poor appetite control [63], a concept underpinned by recent research into the “gravitostat” model of body weight feedback and control. In this model, osteocytes may be capable of detecting changes in body mass and affecting appetite in order to maintain a set body weight. Reduced physical activity/increased time spent sitting may reduce the effectiveness of this feedback system leading to increased appetite, overconsumption of food, and weight gain [64, 65]. In addition to these effects of activity on appetite control, decreases in muscle mass, as a result of reduced activity, may result in increased appetite as a consequence of the protein leverage model of appetite regulation [66]. This model hypothesizes that a lower proportion of protein in the diet, potentially due to overconsumption of ultra-processed foods (UPFs), leads to compensatory increases in energy intake in an attempt to maintain a higher absolute protein intake [67]. Thus, a cycle of muscle loss, increased appetite, and fat mass gain may be perpetuated. Reduced activity may also lead to poor sleep duration and quality [68, 69] which also has the potential to affect appetite and subsequently weight control [70–72] with the possibility of further loss of muscle mass [73].The relevance of these concepts will be further discussed later in this article.

### Mechanisms of muscle maintenance and loss

As alluded to above, due to the process of aging which is accelerated by disuse, skeletal muscle displays features of plasticity, enabling growth and decrements over the life course in response to, amongst other things, the stimulus of physical activity [74]. The driving force behind these changes in mass is the equilibrium between MPS and muscle protein breakdown (MPB) with net increases in MPS resulting in increases in muscle size [75]. Both weight bearing or RE and, more acutely, the ingestion of high-quality protein rich in essential amino acids (EAA) (particularly leucine) are potent stimuli of MPS [76].

The mammalian target of rapamycin complex 1 (mTORc1) is a key regulator of MPS and muscle protein turnover. mTORc1 regulates protein synthesis via activation of the eukaryotic initiation factor 4E-binding proteins (4E-BPs) and p70 S6 kinase 1 (S6K1) [77]. This results in increased translation efficiency and capacity of mRNA leading to increased protein synthesis. Resistance exercise activates upstream signaling of mTORc1 to increase MPS and muscle hypertrophy of type II fibers [78]. Mechanical loading of skeletal muscle may be key in mediating mTORc1 stimulation via mechano-sensing proteins; however, exercise-induced muscle damage and metabolic stress may also have a role to play. The direct mechanisms from RE stimulus to mTORc1 activation are yet to be elucidated [79]. Similarly, leucine activates mTORc1 via an amino acid–sensing pathway, perhaps via dissociation of Sestrin1 from the GATOR2 complex, to synergistically enhance RE-induced MPS [80, 81]. Given the importance of this pathway in enabling MPS, any perturbations in the process (e.g., social isolation and reduced PA) may culminate in catastrophic losses of muscle mass.

Furthermore, older adults experience a phenomenon known as anabolic resistance, a diminished response to the MPS-stimulating effects of physical activity and protein ingestion [82–84], which is believed to be a primary contributor to the development of sarcopenia. For example, it has been reported that older compared to younger men (mean 71 years vs 22 years) require approximately twice the amount of high-quality protein (0.60 vs 0.25 g/kg lean body mass) to maximally stimulate MPS [84]. For the average older adult, this may be approximately 40 g of protein per meal [85]. Similarly, exercise-induced MPS rates are attenuated in older compared with younger individuals [86] meaning that greater durations or intensities of exercise may be needed to maintain muscle in older individuals. In contrast to expectations, rather than a reduction in mTORC1 activation with aging, rodent studies have illustrated that mTORc1 may actually be hyper-activated in older, sarcopenic individuals [87], suggesting the presence of mTORC1 resistance with age.

Anabolic resistance and the subsequent muscle loss is multifactorial and associated with an often interrelated decrease in physical inactivity, inadequate dietary quality, increased adiposity, increased inflammation, dysregulated hormones, and other comorbidities [88]. The age-associated increase in inflammation, or inflamm-aging, is highlighted by chronic elevation of inflammatory biomarkers such as interleukin-6 (IL-6), tumor necrosis factor alpha (TNF-α), and C-reactive protein (CRP) amongst others [89]. Indeed, sarcopenic populations have been shown to have higher levels of CRP compared to age-matched controls without sarcopenia [90]. Inflammation has been shown to upregulate catabolic pathways and downregulate anabolic pathways, thereby reducing net MPS [91]. For example, in vitro studies have reported that TNF-α inhibits myogenesis and upregulates nuclear factor-kappa beta (NF-κβ), a key transcription factor in skeletal muscle atrophy [92, 93]. In the context of the current COVID-19 pandemic, the importance of inflammatory cytokines is becoming more apparent with increased levels of proinflammatory cytokines such as IFN-α, IL-6, IL-12, IL-17, IL-18, IL-33, TNF-α, CRP, and MCP1 (known as the “cytokine storm”) observed in patients with severe COVID-19 [94]. These not only contribute directly to tissue damage [95] but may also contribute to sarcopenia by blunting MPS [90] during immobilization and beyond.

Aging is also associated with a decrease in hormones that regulate muscle mass such as growth hormone, dehydroepiandrosterone, testosterone, insulin-like growth factor I (IGF-I), and estrogens [96]. Pro-inflammatory states such as those observed in severe COVID-19 infection are also associated with reductions in hormones such as testosterone [97] and IGF-I [98] and are believed to contribute to reductions in muscle mass [99, 100]. Therefore, it is not simply the bed rest, which culminates in wasting, but elevated proinflammatory and reduced anabolic agents exerting direct effects on muscle catabolism. During puberty, these anabolic hormones are increased leading to increased height, muscle mass, and sex-specific phenotypes [101]. Therefore, the age-related decline in these hormones, particularly testosterone in males, may regulate the decline in muscle mass with age. For example, long-term testosterone replacement therapy has been reported to increase lean body mass, muscle strength, and power in older men [102, 103]. Testosterone regulates MPS via the androgen receptor [104] and its administration has been reported to augment anabolic signaling and MPS in response to RE in older males, suggesting a role in reducing anabolic resistance to RE [105]. However, testosterone administration alone in older adults may be unable to fully reduce the age-related decline in MPS [106].

Low levels of physical activity and poor dietary habits, which may be more prevalent during COVID-19 confinement [25], are associated with obesity and a range of comorbidities including metabolic syndrome (MetS), T2DM, and CVD [107–109]. Obesity, particularly abdominal obesity, and the aforementioned comorbidities are also associated with increased levels of inflammation and dysregulated anabolic hormones, which may further exacerbate anabolic resistance [110]. Indeed, these comorbidities and lifestyle factors are typically associated with low muscle mass and sarcopenia and may also contribute to anabolic resistance [111]. For example, the PI3K/Akt signaling cascade is a key pathway in regulating growth, with its activation, particularly by the anabolic hormone insulin, inhibiting the atrophy-related protein forkhead Box-O1 (FOXO) while also activating mTORc1 [112, 113]. The diminished response to insulin is a prominent phenotype observed in insulin-resistant states such as MetS and T2DM and is also associated with age [108, 114]. In the context of sarcopenia and the current prevalence of T2DM, this has important implications for muscle mass. Indeed, both hyperglycemia and IR are important in the declining muscle mass observed in diabetes [115–117]. Insulin resistance can be induced by extended periods of inactivity/bed rest [44] which may not only put people at greater risk of muscle loss due to social distancing measures but also may lead to greater susceptibility to COVID-19 itself [118].

Indeed, recent reports of confinement during COVID-19 highlight a 33.5% decrease in number of minutes/day of PA and increases in number of main meals and snacking [25] which may make positive energy balance and fat accumulation more likely. Furthermore, increased adiposity in older populations, such as ectopic accumulation of fat between muscle fibers, known as intramuscular adipose tissue (IMAT) has also been shown to impose a significant risk of muscle dysfunction in older adults [119, 120]. Alterations in energy status (such as increased lipid metabolites like diacylglycerols and ceramides) in addition to increased inflammation have been shown to activate protein kinase C theta (PKC-θ), c-Jun-N-terminal kinase (JNK), and inhibitor of kappa B kinase (IKK) [117, 121, 122] resulting in suppressed protein synthesis and increased protein breakdown. Increased adiposity has also been reported as a risk factor for COVID-19 infection and severity such as admission to ICU (adjusted OR 5.39) and the need for IMV (aOR 9.99) [29] thus posing a double risk at this time.

Another mechanism which may contribute to anabolic resistance in older populations is reduced capillarization of skeletal muscle, which may blunt the hypertrophic effect of RE. To illustrate this, Moro et al. [123] demonstrated that amongst a group of older adults (mean age 71 years) participating in a 12-week RE program, those with lower baseline muscle capillarization did not experience muscle hypertrophy, whereas participants with higher muscle capillarization did. As mentioned previously, IMAT may also contribute to the reduced hypertrophic response seen in aging muscle as Marcus et al. [124] demonstrated that in older adults (mean age 73 years) performing 12 weeks of RE, only those with low IMAT showed improvements in muscle quality [124]. Sarcopenia is very much a “chicken or egg” scenario as it is unknown if these age-related changes precede sarcopenia and frailty, leading to decreased activity or if chronically reduced activity results in dysregulated anabolic/catabolic signaling [125]. However, what is well established is that regular exercise throughout the lifespan reduces the severity of sarcopenia and its associated comorbidities [88] as well as being associated with improved immune function [126–128]. Older individuals are already compromised in terms of muscle mass, compared with younger counterparts and are therefore, relatively speaking, at a significantly elevated risk of muscle loss if unexpected perturbations are encountered. Therefore, a number of factors related to the COVID-19 pandemic may further contribute to this loss of muscle mass and function with aging and significantly impact on the health span of an aging population.

### Food access, dietary intake, and energy balance

Changes in access to food, for example due to temporary shortages because of panic buying or due to less frequent visits to grocery stores, as a result of government restrictions and/or fear or anxiety of possible infection [129], may lead to changes in food choices and diet quality. These dietary changes, along with changes in appetite regulation (which will be discussed later), have the potential to take two, opposing directions: that is, scenarios involving positive and negative energy balance are both possible. Indeed, recent research has reported that 30% of respondents to a COVID-19-related survey reported weight gain (mean 3.0 kg) and over 18% reported weight loss (mean − 2.9 kg). There was a tendency for participants with overweight and obesity, and subjects over 36 years, to gain weight, whereas underweight participants tended to lose weight [130]. This may indicate that confinement during COIVD-19 may exacerbate over- or undereating in different individuals depending on pre-existing tendencies.

On one hand, positive energy balance may result from an increased reliance on UPFs and convenience foods due to both their longer shelf life and an increase in emotional/stress eating [129, 131]. Indeed an increase in the intake of such foods (specifically, potato chips and sugary drinks) has been observed amongst children living through lockdown in Italy [43]. The same study also reported an increase in average number of meals of 1.15 per day. Further research from the Italian lockdown reported that 46.1% of respondents felt they ate more during confinement and in particular, high-calorie “comfort foods” such as chocolate, ice cream, desserts, and salty snacks, which was mostly attributed to higher levels of anxiety [132]. This increased frequency of eating and reliance on high-calorie UPFs can potentially affect muscle mass in two ways. Firstly, diets higher in UPFs tend to be lower in quality, specifically, lower in protein which may reduce the capacity to stimulate muscle growth [39]. Secondly, such diets can lead to an increase in calorie intake, leading to a positive energy balance that may result in body fat gain [39, 133]. Excess body fat can contribute to muscle loss by reducing ease of locomotion: an individual with sarcopenia and elevated fat mass (sarcopenic obesity [SO]) will have difficulty in moving due to low muscle strength and the excess weight of the fat mass, resulting in decreases in non-exercise activity thermogenesis (NEAT) and physical activity [134]. This can lead to further weight gain, exacerbating the cycle. Excess fat mass is also known to lead to low-grade systemic inflammation which can result in IR [135] and obesity-related metabolic diseases [136] and contribute to sarcopenia [100], as previously discussed. A further potential complication of this confinement-induced obesity is the increased risk of COVID-19 infection and severity [28, 137] with severe obesity being associated with admission to ICU (adjusted OR 5.39) [29].

Contrary to the potential for weight gain, there is also a risk of reduced access to and/or means to buy enough food to maintain weight and/or adequate nutrition [32], which could lead to weight loss as an alternate outcome. As of 2016, 21% of UK adults (16 years and older) were classified as marginally to severely food insecure, with a high proportion of unemployed or those in low-income households reporting difficulties in meeting food needs [138]. For older adults, this food insecurity may be amplified by a reluctance to leave home to go grocery shopping, due to their recognition as an “at-risk” population [46, 139] coupled with a lower use of online/delivery-based grocery services [140]. Thus, there is also a risk of reduced food intake which may lead to weight loss. As approximately 25% of body mass lost during weight loss can be attributed to fat-free mass (including muscle mass) in young and healthy individuals [141], undesired weight loss may further contribute to the acute loss of muscle mass in older individuals [142] during the COVID-19 pandemic.

This situation of altered access to food may be further compounded by financial issues due to the pandemic-associated restrictions. The UK Office for National Statistics has reported that almost one quarter (23%) of surveyed adults have admitted that their household finances were affected with the majority being worried about their income [143], although older individuals in receipt of a pension may not be affected.

### Impaired sleep, stress, and anxiety

While not immediately apparent, psychological factors, sleep, and anxiety may play a considerable role in the loss of muscle during a pandemic. This can be due to their effects on health behaviors such as eating habits and physical activity, as well as changes in metabolic pathways related to maintenance of muscle mass. Enforced quarantine and even isolation due to social distancing measures during the COVID-19 pandemic have the potential to cause considerable emotional issues. Indeed, a recent COVID-19-related study from Italy reported poor sleep quality in 57.1%, high anxiety in 32.1%, and high distress in 41.8% of participants [144]. Another recently published study has also reported symptoms of COVID-19-related post-traumatic stress disorder (PTSD) in as many as 29.5% of respondents [145].

Eating is recognized as a coping mechanism for dealing with stress and emotions [146, 147]. Those with the lowest scores for emotional and stress-related eating, as measured by the Eating and Appraisal Due to Emotions and Stress (EADES) Questionnaire, are up to 13.38 times more likely to present with overweight or obesity, compared with those with the highest scores [148]. With many people experiencing negative emotions and stress due to lockdown, there is also an increased likelihood of stress eating and overconsumption. Combined with hyperpalatable UPFs [133, 149, 150] frequently purchased in anticipation of times of food shortage [151], overeating becomes an even more probable consequence.

Stress is also associated with sleep disturbance, shorter sleep duration, nighttime awakening, and insomnia [152, 153]. Changes in daily schedules due to confinement may also contribute to poor sleep quality due to disruptions in circadian rhythms which may already be disrupted in older adults [154]. Indeed, recent data from individuals quarantined during the COVID-19 outbreak in China reported anxiety correlated with stress resulting in reduced sleep quality [155], while an Italian study [156] reported lower sleep quality despite participants spending more time in bed.

Both stress and sleep curtailment can contribute directly to muscle loss through changes in key chemical messengers in metabolic pathways related with muscle mass. Short-term, modest sleep curtailment (from 8 to 6 h/night) has been shown to increase proinflammatory cytokines such as IL-6 and TNF-α [157] which are associated with muscle loss [99]. Sleep loss is also associated with dysregulation of hormone secretion, such as elevated cortisol resulting from 2 nights of 4 h of sleep [158] or reduction in testosterone levels by 10–15% resulting from 8 nights of 5 h of sleep [159]. Hypercortisolemia is reported to increase MPB, which is amplified by inactivity [160], a potentially likely situation during both COVID-19 confinement and hospitalization.

Reductions in sleep duration and/or quality can also lead to changes in appetite and hunger [71]. Recent data from populations during COVID-19 confinement indicates that as many as 57.1% of some cohorts experience poor sleep quality [144] and other surveys have reported as many as 46.1% of respondents were consuming more high-calorie foods [132]. It is believed that at least some of these effects are caused by changes in satiety hormones such as leptin (which reduces appetite) and ghrelin (which increases food intake). For example, sleep deprivation studies have shown that after only 2 nights of 4 h sleep each, leptin levels can drop by 18% and ghrelin can increase by 28%, resulting in a 23% increase in hunger with a preference for high carbohydrate foods [70]. Similarly, Yang et al. observed that after only one night of modest sleep curtailment, food cravings, food reward, and selected portion sizes of food increased in healthy women [161]. Such dysregulation of appetite control coupled with access to hyperpalatable UPFs with low satiety value [133], and reduced activity levels creates a perfectly obesogenic storm. As previously discussed, excess adipose tissue can contribute to muscle loss through impaired locomotion and metabolic/hormonal dysregulation such as chronic inflammation and IR [100, 135, 162].

**Fig. 2:**
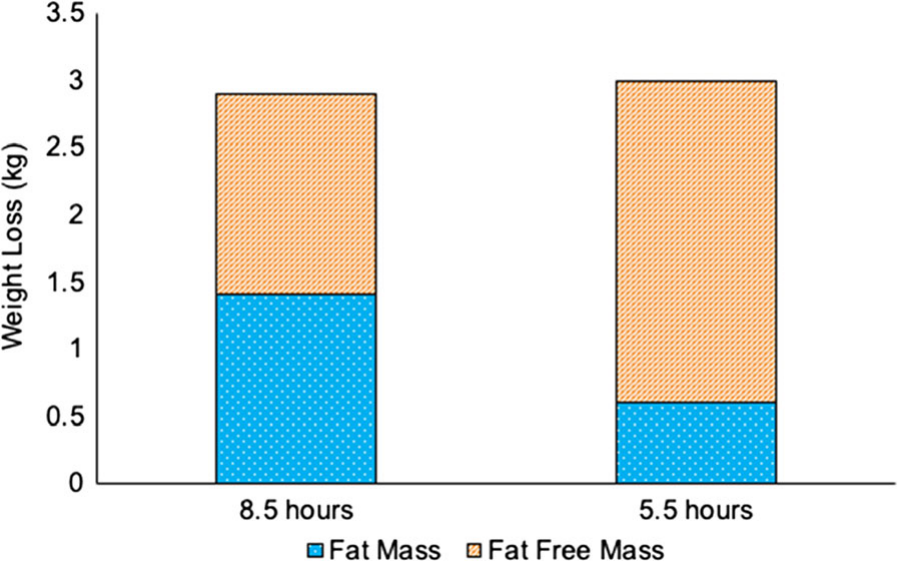
Composition of changes in body weight during calorie restriction under normal and restricted sleep conditions. Under conditions of restricted sleep (5.5 h), greater weight was lost as fat-free mass (including muscle) and less body fat was lost, compared to conditions of sufficient sleep (8.5 h). Adapted from Nedeltcheva et al. (2010) [73]

It has been shown that COVID-19 confinement can also result in weight loss in certain individuals and, when combined with reduced sleep, may also contribute to muscle loss. Nedeltcheva et al. [73] showed that in a calorie deficit, individuals who slept 5.5 h lost 55% less fat and 60% more fat-free mass, compared to those who slept 8.5 h over 2 weeks (Fig. 2). Thus, the problem of weight loss resulting in lean mass loss in the older individuals [142] may be exacerbated by poor sleep during the pandemic.

Another mechanism by which stress, anxiety, and impaired sleep may lead to muscle loss is through their effects on health behaviors. A study by Strine et al. [163] highlighted that people with frequent sleep insufficiency were significantly more likely to engage in adverse health behaviors including smoking, physical inactivity, and heavy drinking. These results were replicated by Walsh et al. who also reported that those suffering with depression, anxiety, and stress were less likely to engage in health-promoting behaviors such as consuming vegetables and eating breakfast [164]. It could be speculated that lockdown-induced low mood and stress could make it less likely for people to engage in health behaviors necessary for the maintenance of muscle mass, namely exercise, and research on how stress impairs efforts to exercise has been reported previously [165]. Poor sleep duration and quality may also result in higher levels of perceived stress and anxiety, thus fueling a vicious cycle of sleep disturbances and stress [166, 167].

Critically, even after the lifting of quarantine restrictions, psychological distress may result in some individuals continuing to avoid enclosed places where large groups of people gather or even outdoor public spaces [129]. This is particularly relevant in a post-COVID-19 situation as access to gyms and fitness centers along with outdoor recreational spaces such as sports grounds and public parks may be vital to efforts to improve muscle mass, strength, and physical fitness as well as for improving social interaction and engagement [168–170].

### Reduced sun exposure and vitamin D

Vitamin D (specifically the active form 1,25-dihydroxycholecalciferol) has historically been linked to bone health. However, there are multiple studies that have shown poor vitamin D status to be associated with multiple chronic diseases [171] and reduced muscle mass [172]. This may be especially important during the current COVID-19 pandemic due to lockdown measures that lead to people experiencing less direct sunlight, thus impacting negatively on vitamin D synthesis [173].

Vitamin D plays an important role in the regulation of muscle contraction, with deficiency altering sarcoplasmic calcium handling leading to prolonged muscle relaxation [174]. This may also impair mitochondrial energetics, and indeed correcting vitamin D status has been shown to improve mitochondrial oxidative function in humans [175]. Similarly, Dzik et al. [176] showed that vitamin D supplementation relieved lower back pain, reduced cytosolic superoxide dismutase (SOD) and glutathione peroxidase (GPx) activities, and decreased 8-isoprostanes and protein carbonyls in patients’ multifidus muscle.

In vitro studies have shown that vitamin D can enhance insulin signaling via the Akt/mTORc1 pathway, and stimulate protein synthesis to a greater extent than when cells were exposed to insulin plus leucine alone [177]. Increased phosphorylation of the insulin receptor was also observed, together with an upregulation of the vitamin D receptor (VDR). More recent work in mice has shown that muscle-specific deletion of VDR leads to significant changes in body composition, resulting in greater percentage of fat mass and reduced lean tissue [178]. These physical changes were accompanied by functional alterations, including decreased time spent running, lower speed, and lower grip strength (reflecting chronic and acute types of effort) [178]. Indeed, it has been shown that vitamin D decreases the expression of myostatin, a negative regulator of muscle mass [179], potentially explaining the negative consequences of vitamin D deficiency on muscle size. These findings offer some potential mechanistic insight into the studies that have shown an association between vitamin D status and muscle mass and strength in older people [180, 181].

Much of the data regarding vitamin D status and muscle status in humans is derived from observational studies; however, there have been several insightful randomized controlled trials examining the effect of vitamin D repletion on muscle function. Burns patients are at increased risk of hypovitaminosis D and therefore present a novel opportunity to examine restoration of vitamin D status. In 15 adults with thermal burns, quarterly intramuscular injections with 200,000 IU vitamin D and daily oral calcium led to a significant increase in quadriceps strength when compared to baseline values, showing a direct effect of vitamin D supplementation [182]. While a recent systematic review and meta-analysis suggested a small, non-significant (*P* = 0.06) increase in muscle strength, subgroup analysis showed improvement with doses of > 1000 IU/day, > 3-month treatment duration, and in participants with a baseline vitamin D concentration of < 30 ng/mL [183]. Thus, improvements may not be seen in those individuals who have an adequate vitamin D status. Data presented at the 21st European Congress of Endocrinology also suggests that more substantial benefits from vitamin D on muscle tissue are observed when combined with increased protein supplementation [184].

Vitamin D deficiency (25(OH)-vitamin D level < 20 ng/ml) has also been suggested as risk factor for COVID-19 infection [185] and may contribute to its severity through its association with increased proinflammatory cytokines [186]. The prevalence of vitamin D deficiency amongst older adults may be as high as 65% in some groups in the UK [173, 187]. Additionally, older adults with reduced mobility/muscle function and those who spend most of the day indoors are at a greater risk of deficiency [188, 189]. Therefore, deficiency may play a considerable role in not only the etiology of sarcopenia but also the severity of COVID-19 during lockdown, when sun exposure may be further reduced in the self-isolating elderly or those hospitalized due to COVID-19.

## The relationship between muscle loss and chronic lifestyle conditions

**Fig. 3:**
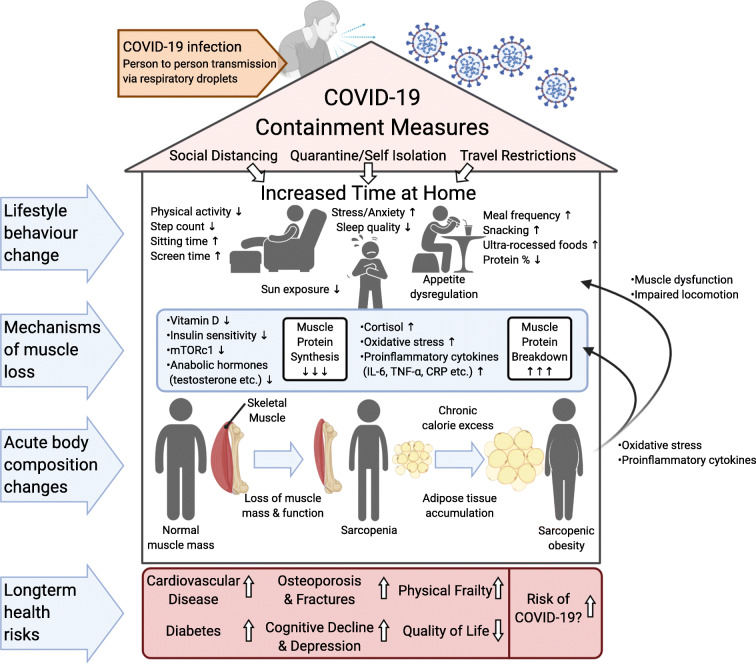
Summary of potential effects of government restrictions on lifestyle behaviors and the mechanisms by which they can lead to reduced muscle protein synthesis and increased muscle protein breakdown resulting in muscle loss. The development of sarcopenia, or in the presence of caloric excess, sarcopenic obesity, is associated with a significantly increased risk of multiple comorbidities, some of which may also increase the risk of COVID-19 infection and severity. COVID-19 severe acute respiratory syndrome coronavirus 2, mTORc1 mammalian target of rapamycin complex 1, IL-6 interleukin-6, TNF-α tumor necrosis factor alpha, CRP C-reactive protein

Muscle loss is associated with a number of metabolic, physiologic, and psychologic/cognitive pathologies. It is likely that the development of these pathologies is related to not only the loss of muscle mass, but also an increased prevalence of adipose tissue and particularly visceral adipose tissue (VAT) and IMAT as observed in sarcopenic obesity [190, 191]. Visceral adipose tissue is independently associated with the incidence of CVD, even after adjusting for other clinical risk factors such as T2DM, total cholesterol, smoking, hypertension, and body mass index (BMI) [192], which may be a result of higher levels of proinflammatory cytokines produced in VAT [193]. These may further contribute to the progression of SO through their association with reduced muscle mass and strength [56, 99, 194]. Furthermore, IMAT has also been shown to increase as we age and can cause both a reduction in the physical capacity of skeletal muscle [195] and an increase in local and systemic inflammation, again through the secretion of proinflammatory cytokines [119, 196]. The comorbidities associated with this loss of muscle mass and increase in VAT and IMAT and their potential consequences in relation to COVID-19 infection and severity will be discussed briefly here (Fig. 3).

### Cardiovascular disease

Low muscle mass is associated with greater risk of and mortality from CVD [197, 198]. In a population of 6451 patients with CVD, Srikanthan et al. demonstrated that both disease-specific and all-cause mortality were significantly greater in those with lower compared to higher muscle mass, regardless of fat mass, indicating high muscle mass may play a protective role in CVD [199]. Sarcopenia is also independently associated with non-alcoholic fatty liver disease (NAFLD) and T2DM, both of which are risk factors for CVD [200, 201]. A recent systematic review reported that gait speed and handgrip strength, both of which are used in some definitions of sarcopenia [1] and are dependent on muscle function, are associated with CVD mortality and in many of the included studies, this association was independent of traditional risk factors such as smoking and dyslipidemia [202]. Potential mechanisms for this elevated risk of CVD in sarcopenia are increased LDL cholesterol, blood pressure, oxidative stress, proinflammatory cytokines, and decreased insulin sensitivity associated with sarcopenic changes in muscle tissue [100, 203, 204]. These factors are known to contribute to the development of atherosclerotic plaques, which is a key process in coronary heart disease [205]. Of particular concern is the elevated incidence of COVID-19 in individuals with comorbidities such as hypertension and diagnosed CVD, which were observed in up to 31% and 15% of COVID-19 patients, respectively [206]. In the same cohort, it was observed that hypertension and CVD are even more prevalent in patients requiring ICU admission, 58% and 25%, respectively. With such significant associations between reduced muscle mass, CVD, and the risk of severe COVID-19 infection, public health authorities need to carefully consider measures to reduce the potential declines in muscle mass that can precede CVD. This will be vitally important if individuals are to improve healthspan and reduce risk of mortality from COVID-19, should second wave predictions become a reality [47].

### Diabetes

In the English Longitudinal Study of Ageing, participants with obesity and with handgrip strength below the threshold of weakness (a proxy for sarcopenia/dynapenia) were over 3.5 times more likely to develop T2DM over 6 years [207]. Skeletal muscle is the largest insulin-sensitive tissue in the body and accounts for 80% of glucose uptake under hyperinsulinemic, euglycemic conditions, and IR of this tissue is a key process in the development of T2DM [113, 208]. Thus, lower levels of muscle mass, as observed in sarcopenia, may lead to a reduced capacity for glucose disposal in older adults. Older age and sarcopenia are also associated with IMAT accumulation [120] which may reduce insulin sensitivity [209, 210]. IMAT may also contribute to a proinflammatory state through elevated levels of cytokines such as IL-6, CRP, and adipokines such as leptin as well as reduced levels of anti-inflammatory and insulin-sensitizing adipokines such as adiponectin [211]. It should be highlighted that this may also contribute to further muscle loss due to impairments in regulation of protein metabolism/synthesis, thereby maintaining a vicious cycle of worsening sarcopenia and IR [212]. Of further concern, a recently published meta-analysis showed the pooled prevalence of diabetes in COVID-19 was 9.8% and it was significantly associated with both risk of severity and mortality with pooled odds ratios of 2.75 and 1.90, respectively [118]. A study of COVID-19-associated mortality in Italy also observed diabetes in 36% of deaths [213]. Furthermore, an increased incidence of fasting glycemia and acute-onset diabetes has been reported among patients with COVID-19 leading to the hypothesis that it may cause “new-onset” diabetes in patients without diabetes [214]. This further highlights the links between muscle loss, metabolic perturbations, and increased risk of COVID-19 during this pandemic.

### Cognitive decline and depression

Sarcopenia is independently associated with cognitive impairment (declines in cognitive functions such as verbal memory, working memory, interference control, and processing speed) and depression [11, 215–217]. Many of the risk factors associated with cognitive impairment such as low levels of exercise, reduced anabolic hormones, malnutrition, and low-grade chronic inflammation are also known causes of sarcopenia [218]. Cognitive function is strongly associated with the integrity of the neural connection pathways needed for muscle movement and coordination [219] and this may highlight the importance of including measures of muscle function/strength and not just size in definitions of sarcopenia. While studies have established an association between depressive symptoms and sarcopenia, this seems not to be related directly to muscle mass and instead is related to reduced muscle strength and function [10]. To complicate this relationship further, late-life depression can lead to further declines in cognition [220] and is also associated with reduced physical activity and increased sedentary behavior [221] which may further exacerbate sarcopenia. Higher levels of physical activity and lower levels of sedentary time are consistently associated with better mood scores [222, 223] but current social distancing and self-isolation measures will likely lead to greater social isolation in some individuals which is associated with lower levels of physical activity [224, 225]. Thus, social isolation resulting from COVID-19 social distancing measures may have significant implications on physical activity levels, mental health and well-being, feelings of isolation, depressed mood, and muscle loss.

### Osteoporosis and risk of fractures

Low muscle mass and strength are associated with bone mineral density abnormalities and osteoporosis in older men and women [226, 227]. In a sample of 679 middle-aged and elderly male Europeans, those with sarcopenia were 3 times more likely to have osteoporosis compared with those with normal muscle mass, defined as relative appendicular skeletal muscle mass ≥ 7.26 kg/m^2^ [228]. As osteoporosis is frequently associated with fracture risk, it is not surprising that sarcopenia is also associated with an elevated risk of fractures [8, 229]. The process of bone remodeling is carried out by bone cells such as osteoblast which help with the formation and repair of bone, osteoclasts which break down bone, and osteocytes which have a mechano-sensitive function which can detect the mechanical forces of muscle movement [230, 231]. Decreases in the physical stimulus of muscle contraction, such as could be induced by inactivity or hospitalization during the COVID-19 pandemic, lead to a reduction in hormones such as testosterone, estrogen, or growth hormone [232–234], and increases in proinflammatory cytokines, such as interleukin-1 (IL-1), IL-6, and tumor necrosis factor-alpha (TNF-α) [235]. Such conditions have been shown to lead to reduced osteoblast and enhanced osteoclast activity which can result in osteoporosis [236]. Thus, bone mass, size, and density are influenced by exercise, similarly to how muscle size and quality can be affected by regular activity [237]. With the significant decreases in PA during the COVID-19, there is an increased risk of falls-related fractures with associated morbidity and early mortality as a consequence [238]. A program of prehabilitation and rehabilitation for older adults may therefore be prudent.

### Frailty risk of falls and quality of life

While there is no consensus definition of frailty [239], it is considered to be responsible for disability independently of clinical and subclinical disease. The central features of frailty include weakness, decreased endurance, and slowed performance resulting from a cumulative decline across multiple physiologic systems with advancing age [239]. The loss of muscle size and, more importantly, loss of strength and function associated with sarcopenia may contribute to the development of frailty [240, 241] and as such the diagnosis of sarcopenia may be a useful predictor of frailty [242, 243]. An additional consequence of the physical decline resulting from sarcopenia/frailty is an increased risk of falls [244–246] and it should be noted that falls are the leading cause of fatal and non-fatal injuries in older individuals [247]. With older individuals being more susceptible to severe COVID-19 and more likely to require admission to ICU [248, 249], they may be at a greater risk of suffering further muscle loss due to hospitalization, further compounding their degree of frailty.

As illustrated here, sarcopenia is associated with multiple other debilitating pathologies which can greatly add to the disease burden of older adults and reduce their quality of life (QoL). In a population of over 500 community-dwelling older adults, Beaudart et al. [244] reported that even after adjustment for multiple confounders such as age, BMI, and number of comorbidities, participants with sarcopenia had a worse physical health-related QoL, were more frail, were at higher risk of falls, had more difficulty with achievement of activities of daily living, and were also more dependent on others for household than those without sarcopenia. As autonomy in activities of daily living plays a role in multiple bio-psycho-social factors of life in the elderly, reduced autonomy can contribute to reduced quality of life and well-being [250, 251]. COVID-19 may contribute to this reduced autonomy by imposed isolation measures and reduced time spent outdoors as well as through sarcopenia, induced by inactivity and/or hospitalization.

### Mortality

While the association between reduced muscle mass and multiple other comorbidities is apparent, it should also be highlighted that sarcopenia is itself associated with greater risk of death in multiple elderly populations. Of particular concern is the potential risk of mortality that sarcopenia may confer on older patients in acute hospital care, potentially as a result of COVID-19 infection. Sipers et al. reported that in a hospitalized geriatric population, the presence of sarcopenia was significantly associated with up to 4.3 times greater 2-year mortality compared to patients without [252]. In fact, the detrimental effects of reduced muscle mass and strength may be further augmented by elevated fat mass as seen in SO which is also associated with greater all-cause mortality [253, 254]. While there is no consensus definition of SO, the use of measurements of visceral fat area seems to be particularly strongly associated with increased mortality risk compared with those without SO (HR = 2.54) further highlighting the detrimental health effects of this pattern of fat distribution [255]. Similarly, lower rates of all-cause mortality have been observed in older individuals with high muscle mass and low fat mass [199].

### Immune function and risk of COVID-19 infection

While we have briefly described some of the long-term risks of muscle loss and other body compositional changes here, it should also be highlighted that these changes may also result in a more immediate problem, that being susceptibility to, and risk of more extreme presentation of, COVID-19. Early reports from multiple centers worldwide have highlighted that individuals with cardiometabolic comorbidities including T2DM, CVD, and also obesity are at greater risk of COVID-19 infection [137, 256, 257], more likely to require acute care such as IMV [28, 29, 257], and at a greater risk of death [258, 259]. This has led government bodies such as the Center for Disease Control and Prevention (CDC) to advise that individuals with these conditions (all of which have been associated with sarcopenia) are amongst those at greatest risk from COVID-19 [139].

Skeletal muscle is recognized as an endocrine organ [260] which secretes cytokines (known as myokines) such as IL-6 [261], IL-7 [262], and IL-15 [263] in response to physical activity. Changes in circulating levels of these myokines, resulting from the various aspects of the aging process including increased inflammation and sarcopenia, are believed to play a role in the age-associated impairment of the immune response (immunosenescence) [264]. This highlights another mechanism by which sarcopenia may impact the health of older adults. Accordingly, lower levels of activity are associated with reduced immune function. For example, in a sample of older adults (60–79 years), sedentary individuals (2000–4500 steps/day) showed lower frequency of naive T cells and a higher frequency of memory T cells which is indicative of impairments in immune responses or immunosenescence, compared with physically active individuals (10,500–15,000 steps/day) [127]. Higher levels of physical activity may therefore be useful for maintaining immune function in older adults. In a sample of older men (65–85 years), those who regularly engaged in moderate or intense exercise demonstrated superior antibody responses to the influenza vaccine, resulting in higher percentages of seroprotected individuals, compared with age-matched, sedentary controls [265]. Similarly, a 10-month, moderate-intensity exercise intervention was reported to increase the antibody titer in response to influenza immunization in adults over 65 [266]. Additionally, the relationship between muscle mass and myokines may be bi-directional and changes in myokine secretion due to aging may contribute to anabolic resistance and sarcopenia. For example, reduced secretion of IL-15 may contribute to inflammation-related muscle loss in older adults [267]. The global decrease in PA during COVID-19, as evidenced by reductions in step counts and increases in sedentary activity [24], may contribute to a decline in muscle mass and subsequently immune function. This impaired immune function and proinflammatory status may at least partially explain the higher risk of mortality from COVID-19 experienced by older adults [268].

In light of these data, prevention of the development and progression of these conditions in the general population and already at-risk older individuals [46] should be (and in some cases already is) considered amongst government strategies for the management of the COVID-19 pandemic [269]. Such countermeasures are discussed below.

## Countermeasures to prevent sarcopenia during COVID-19

### Resistance exercise

Exercise should be considered of prime importance in attempting to halt and even reverse the progression of sarcopenia. Multiple studies have shown that RE alone (without any dietary, supplementary, or pharmaceutical assistance) can improve muscle size and strength in older individuals [270–273]. This hypertrophic response may be further augmented by the addition of supplementary protein, amino acids, or high-protein diets [274, 275]; the use of nutritional supplements such as creatine [276]; or the use of therapeutic doses of androgenic hormones such as testosterone, which may be used in some clinical settings [277].

Resistance exercise has also been shown to improve markers of cardiovascular health (e.g., LDL cholesterol and blood pressure) [278, 279], glycemic control (lower HbA1c and improved insulin sensitivity) [280, 281], functional capacity [282, 283], bone mineral density [284, 285], body composition [283, 286], sleep [287], and cognitive performance [288]. It should also be noted that regular exercise is known to improve immune function, a faculty that is particularly important in times of pandemic [126–128]. Thus, the potential benefits of encouraging exercise, and, in particular, RE, at all times and especially during a pandemic, cannot be overstated.

**Fig. 4:**
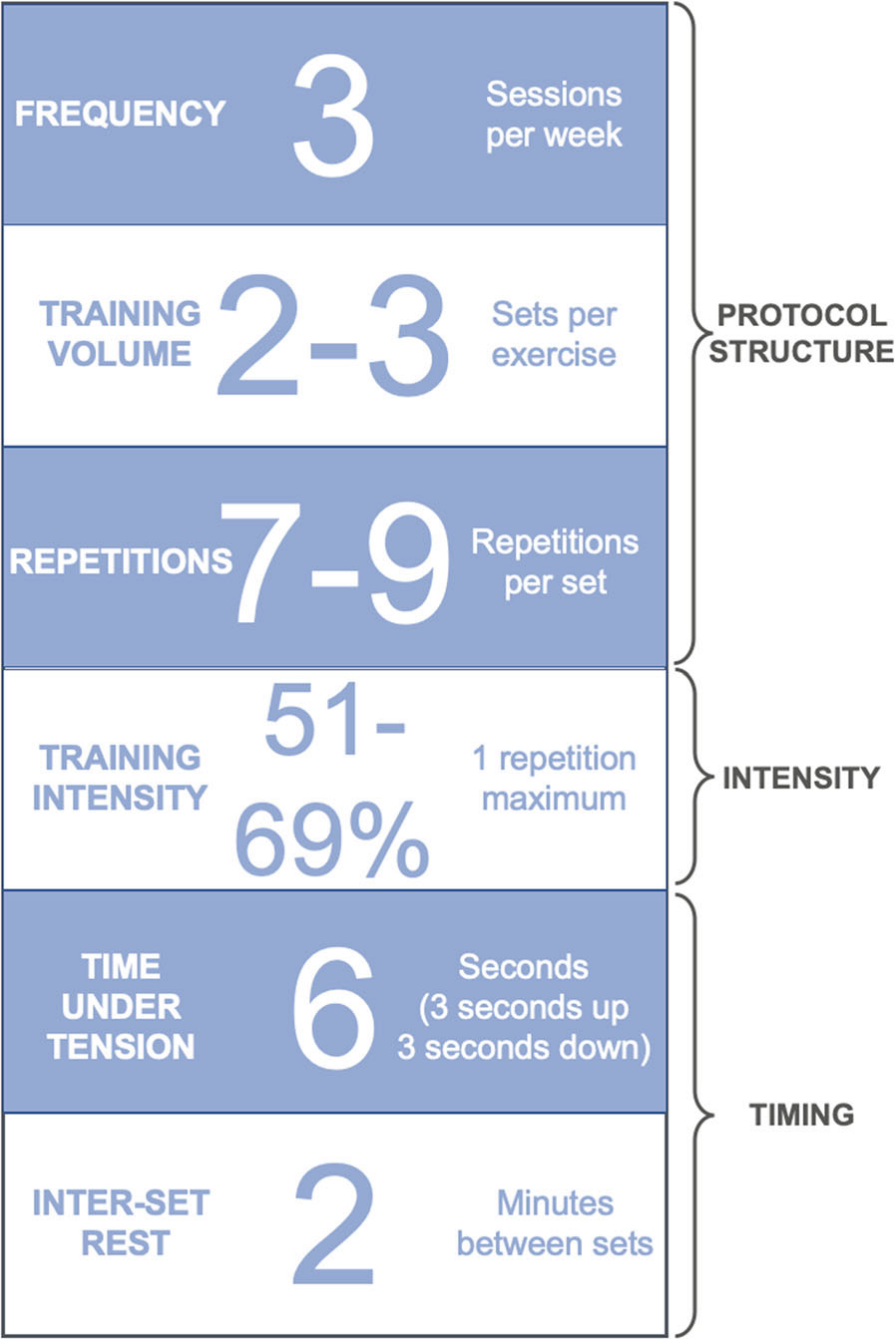
Summary of evidence-based resistance exercise variables reported to improve muscle size in older adults. These figures were calculated using data from a meta-regression of 25 randomized controlled trials. As many variations of training protocols are feasible for muscle gain, this collection of variables should be considered guidelines only and not as a defined training program. Adapted from Borde et al. (2015) [290]

While there are many different ways of implementing RE protocols [289], a meta-regression of data from 25 studies in the older men and women (mean age of 70.4 years, age range 60–90 years) [290] reported that RE to improve muscle size seems to be effective using the following independently computed training variables (Fig. 4).

While implementation of all of these variables may not be feasible during a pandemic, they may act as a useful set of guidelines for developing RE protocols for older adults. Should subsequent waves of COVID-19 enforce future bouts of self-isolation, home-based exercise programs with clear guidance on how to undertake them should be considered, in order to circumvent further periods of inactivity.

Interestingly, recent qualitative research with trainers and older participants in physical activity programs in France highlighted that attendance had fallen even before quarantine restrictions were in place because participants “no longer wanted to have close contact” with the other participants and “no longer wanted to touch the equipment.” However, these same older participants also expressed a need to perform exercise at home [291]; therefore, recommendations for suitable home-based research strategies should be given priority. Even for those who prefer a gym setting, both during and in the aftermath of the COVID-19 pandemic, access to gyms or gym equipment is/will be limited. This may be due to continued social distancing measures and/or measures to protect at-risk groups such as those in older age categories and/or with underlying comorbidities [139]. Therefore, as detailed above, alternatives to free weights and RE machines must be considered and indeed the pandemic may provide an opportunity to engage older age groups in sustainable home-based exercise interventions.

The use of resistance bands is a cost-effective and widely available option that has been proven to be equally effective to conventional (free-weights and machines) RE for improving strength and physical function in older individuals [292, 293]. Band-based and bodyweight training regimes may not offer the resistance offered by adjustable free-weights and RE machines, thus not allowing for the use of training intensities in the 50–70% of 1RM range mentioned previously. However, lower intensity, higher repetition exercise is effective for inducing muscle hypertrophy, as long as momentary muscle failure is achieved [294, 295]. Indeed, at-home training protocols are being developed to maintain physical activity levels and prevent physical decline using minimal equipment, during the COVID-19 pandemic [296, 297], and these should be scrutinized and translated safely from the academic to the home environment. As reduced daily step counts contribute to the loss of lean mass and strength, reductions in insulin sensitivity, and increases in systemic inflammation [298], enabling older people to be more physically active in their own homes will be an essential health measure as we navigate through the pandemic and beyond. Encouraging older adults to walk more, even within their homes and reminding them that physical chores such as cleaning and gardening are relevant and important forms of PA, may be a useful and free initial strategy.

Barriers to participate in RE, including a fear of looking too muscular or a fear of a heart attack or stroke during exercise, have been reported [299]. Given the importance of encouraging engagement in PA, addressing any possible barriers and tailoring progressive PA interventions to ability must be considered. Such barriers can likely be overcome by providing clear information and detailed guidelines to reduce fears and to clarify the health benefits, including preventing muscle deterioration, delaying the disability threshold, reducing risk of falls, building function, feeling more alert, and improving concentration [299]. This is essentially the promotion of PA as a method for maintaining health and well-being into older age, regardless of the climate in which we find ourselves. Furthermore, focusing on modifications in training protocols to improve enjoyment may also be a useful technique for encouraging those at risk of COVID-19-exacerbated sarcopenia to participate in RE, as people are more likely to engage in activities that are enjoyable and avoid activities that are disagreeable [300]. For example, beginning an exercise session with a heavy load and ending with a lighter load has been shown to increase the enjoyment, post-exercise pleasure, and remembered pleasure of a bout of RE [301]. Similarly, in directed exercise settings, focusing on enjoyment in the sessions and using some of the following guiding principles has been shown to encourage affective states and promote exercise adherence over 8 weeks:

- involving participants in exercise selection and program design
- providing positive feedback
- regulating intensity according to participants abilities and wishes
- being transparent about the contents of future training sessions
- increasing training diversity [302]

In addition to principles outlined in the paper above, the following guidelines may also prove useful:

- setting goals and highlighting achievements and progress
- enabling safe, virtual exercise and social domains for those who are motivated by group training
- empowering individuals in the cohorts who are exercising to motivate and to recruit others

While the benefits of RE have been discussed extensively in relation to its ability to improve lean muscle mass and strength, aerobic exercise (AE) should not be overlooked as a potential strategy for the maintenance of healthy muscle mass and function during COVID-19. Chambers et al. [303] analyzed muscle size and adiposity in a population of older individuals (mean age 74 years) who performed, on average, 7 h/week of AE over the previous 52 years. Lifelong AE was shown to attenuate the decline in quadriceps muscle size and isometric strength by ~ 50% in men, compared with non-exercising controls, and higher intensities of exercise were reported to reduce lower body IMAT by ~ 30%. Similarly, Aagaard et al. reported that older individuals (68–78 years) who have engaged in either life-long RE or endurance training have significantly greater maximal muscle strength compared with untrained, control individuals [61] although only strength trained participants demonstrated increased muscle mass. This superiority of RE in comparison to AE is reflected in the widespread use of RE as a key strategy for improving muscle mass and strength in older individuals [270–273].

While AE in isolation may not be as effective as RE in helping to improve or maintain muscle mass and strength in older adults, it may be useful in addition to RE as it can reduce total body fat and IMAT [303, 304], thereby improving muscle function relative to body weight. Such concurrent training strategies have been shown to be more effective than RE or AE alone for increasing gait speed and lower limb strength, and reducing body fat in community-dwelling older adults (mean age 69 years) [304]. Similarly, in a population of untrained, older adults (60–80 years) with abdominal obesity, concurrent training was reported to be more effective for reducing functional limitations and IR than either RE or AE alone [305]. As has been discussed, IR can contribute to anabolic resistance and sarcopenia [117]; therefore, exercise strategies to further reduce IR may be optimal for improving muscle health in the long term. Engagement in AE may offer further benefits by helping to modulate immune response. In an older, sedentary population (61–66 years), 6 months of both AE and RE resulted in increased circulating levels of anti-inflammatory IL-10 and reduced levels of IL-6, CRP, and TNF-α [306], which are all involved in the cytokine storm observed in severe cases of COVID-19 [95]. Interestingly, these improvements were observed to be greater in the AE group.

Current UK exercise guidelines for older adults recommend to accumulate 150 min of moderate-intensity aerobic activity, such as brisk walking, per week [307]. Indeed, with the closure of gyms or suspension of group physical activity programs that may occur in response to a pandemic, walking may be a useful, low-cost, and easily implementable strategy for increasing PA levels. The promotion of walking as physical activity amongst older adults has been shown to be highly feasible and effective for improving physical function, even in those who are functionally limited [308]. Increasing daily steps has also been reported to lead to improved health-related quality of life, better immune function, and improvements in metabolic syndrome and weight maintenance [309]. Walking interventions with a frequency of only 3 days per week have been shown to reduce depression indices in older women [310], which may be especially important considering the increased risk of poorer mental health status during social isolation due to COVID-19. A further benefit to the promotion of walking may also be the increased exposure to sunlight which may help improve vitamin D status [188, 189] and musculoskeletal health.

**Fig. 5:**
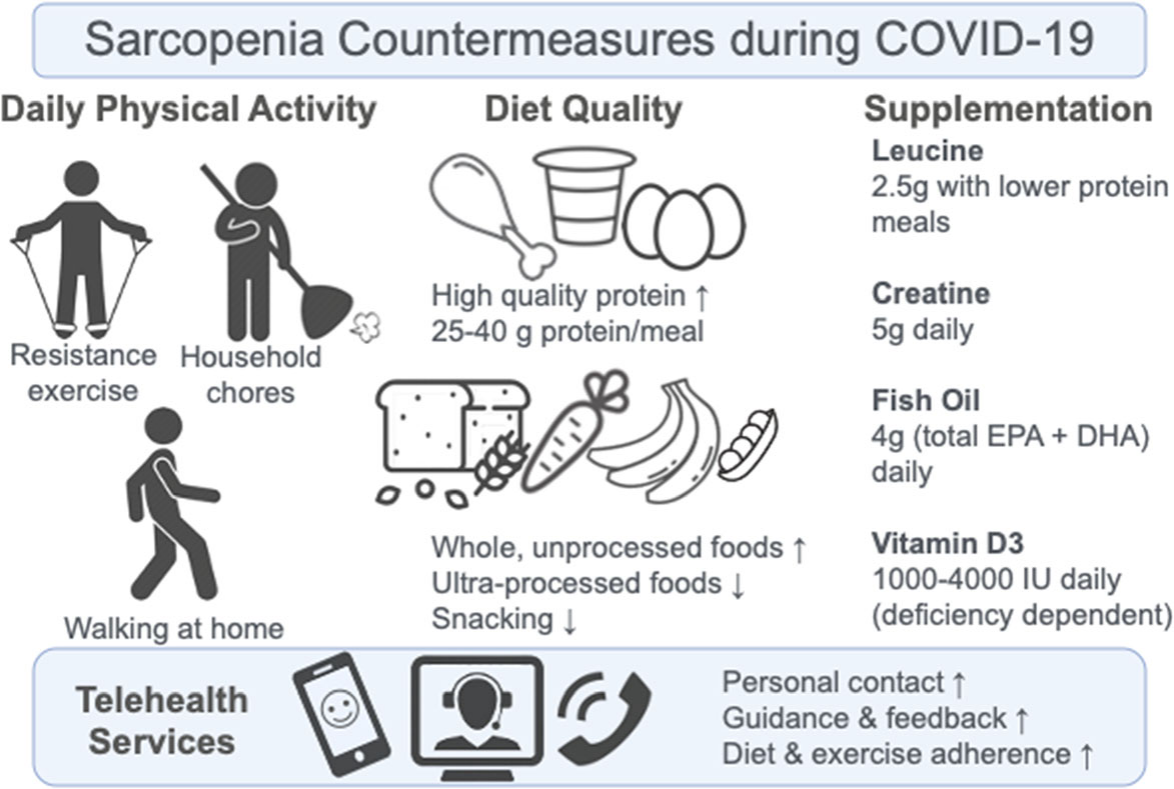
A summary of the physical activity, dietary, and supplement countermeasures that may be useful for preventing the loss of muscle mass and function in both younger and older adults. The inclusion of telehealth services offering regular contact, guidance, and support to such countermeasures may result in greater adherence and positive outcomes

To facilitate these changes in exercise behavior, telehealth services aimed at increasing physical activity, which may involve the use of instructional videos or on-screen interaction with an exercise trainer, should be implemented where possible. There are numerous examples of home-based exercise programs administered through telehealth services that have been beneficial for maintaining physical activity levels and improving health markers such as waist circumference, HOMA index of insulin resistance, and total/HDL cholesterol ratio [311, 312]. A trial of telehealth services, aimed specifically at people with sarcopenia and using remote one-on-one instruction to each participant via video conferencing over 12 weeks, resulted in improvements in muscle mass as well as improvements in functional parameters [313]. Preliminary studies have also highlighted the cost-effectiveness of such telehealth services, and costs may be further reduced if provided as interactive group classes instead of private and if the participant already has their own device (smart phone, tablet, laptop, etc.) [314]. Indeed, group classes may be preferred, particularly in times of social isolation. These results highlight the potential utility of telehealth services for combatting sedentarism and sarcopenia both during and in the aftermath of this pandemic. A list of COVID-19-applicable countermeasures to the loss of muscle mass and function is summarized (Fig. 5) and described in further detail.

### Protein intake

Higher protein intakes can augment the muscle hypertrophic response to RE [275]. The current UK reference nutrient intake (RNI) for protein is 0.75 g of protein per kilogram of body weight per day (g/kg/day) [315]. However, this number does not take into consideration the age-related changes in hormone levels, progressing of sarcopenia, or anabolic resistance previously discussed in this article. More recent research indicates that older adults may need 1.2–1.5 g/kg/day of protein to maintain optimal health and physical function [316, 317]. These articles also highlight the importance of focusing on high-quality proteins, i.e., those that are high in the amino acid, leucine, which is a determinant of both short- and long-term MPS responses in older adults [318]. Encouraging higher protein intakes amongst older adults should be further prioritized as this group has been shown to have protein intakes below the already in adequate RNI with a recent study showing that 35% of participants fail to consume ≥ 0.75 g/kg/day and fewer than 15% consume ≥ 1.2 g/kg/day [319]. Protein intakes may be even lower in older individuals hospitalized due to COVID-19, those with disabilities [320], or, as previously mentioned, amongst those whose diets may depend more on lower quality, lower protein UPFs as a result of food insecurity due to current government sanctions [39, 151]. The importance of encouraging protein intake in older adults in order to prevent muscle loss is further highlighted in the Health, Aging, and Body Composition (Health ABC) Study. Over 3 years, it was observed that community-dwelling adults in the highest quintile of protein intake lost approximately 40% less lean mass (LM) and appendicular LM than those in the lowest quintile of protein intake [321].

Higher meal frequency and higher per meal protein dose are associated with greater lean mass and strength [322] and a more even distribution of protein amongst the main meals is also recommended to maintain muscle mass [316]. For older adults, this even distribution of protein could range from 25 to 40 g of protein, three times a day, focusing on higher quality (leucine-rich) proteins such as meat, fish, dairy, and eggs [316]. As breakfast is traditionally one of the lowest protein meals in the UK, with a mean intake of 12 g in adults over 65 years [323], encouraging inclusion of protein/leucine-rich, lower-calorie foods at breakfast such as low-fat dairy products (Greek yoghurt, quark, cottage cheese, etc.) may be beneficial. Indeed, the use of protein-rich dairy products (both whole food and as protein supplements) has been effective for improving LM and function in multiple RCTs [324–326]. Pre-bed protein ingestion is also thought to be a viable strategy to enhance muscle mass accretion [327]. Therefore, encouraging the addition of a small, high-protein meal before bedtime may further help prevent sarcopenia. Research has also shown that such late-night protein meals (specifically, 48 g of casein protein powder) do not negatively affect sleep [328], thus eliminating the potential catabolic effects of sleep reduction on muscle mass [73].

While protein powders/shakes are frequently used to augment lean mass in scientific research [329], there may be issues with the acceptability of such products or even protein-enriched foods in older populations. Investigations have found that older people are skeptical about such protein-enriched functional foods and barriers to their use in this population can include confusion, distrust, and a perceived lack of personal relevance [330, 331]. A further issue is that older individuals regularly cite price as affecting their food purchasing decisions [332]; the price of protein supplements could result in them being used as meal replacements. This could be speculated to reduce the intake of more nutrient-dense, whole foods and reduce overall diet quality [333]. Thus, focusing on education relating to high-protein, familiar food options (meat, fish, dairy, eggs, legumes, etc.) may be more acceptable. Alternatively, where budget allows, education around high-protein functional foods as well as their relevance for older people may improve their acceptability and use [330] which may be of particular importance for improving muscle mass, at this time.

Finally, it should be noted that higher protein diets are frequently cited as being problematic for kidney health, a concept that likely developed from the use of controlled protein diets (0.8 g/kg/day) in patients with existing chronic kidney disease or reduced glomerular filtration rates [334]. This perception may be common amongst older individuals and may pose a further barrier to the use of higher protein diets to prevent sarcopenia. In individuals with healthy kidney function, however, higher protein intakes do not pose a risk to kidney function [335, 336]. A clinical trial comparing lower with higher protein intakes in individuals with T2DM and nephropathy showed no benefit on glomerular filtration rates from following the lower protein diet, which was also difficult to adhere to [337]. Education about this common misconception may be useful in promoting higher protein intakes.

### Supplementation

There is a broad range of supplements that may be potentially beneficial for improving or at least maintaining muscle mass during the COVID-19 quarantine/social distancing measures. However, a full discussion of their mechanisms of action is beyond the scope of this review, and we will only briefly mention those supplements with the most promise of utility in the current situation.

#### Leucine

The presence of the amino acid leucine in protein sources is a key determinant of the MPS response [318]. As the protein recommendations in this review are considerably higher (up to 40 g per meal, post exercise [85]) than current intakes of protein in the older population, they may be difficult to achieve. Older people may not want to make large changes to their normal eating habits [319] and the satiating effect of protein may make consuming sufficient protein more difficult [338]. Furthermore, high-quality protein sources (meat, fish, dairy, etc.) can be more expensive than other, lower-protein foods, adding another barrier to higher intakes [339]. However, the addition of leucine (2.5 g) to a smaller dose (20 g) of high-quality protein has been shown to enhance MPS under resting conditions in older men [340] and it has also been shown to partially protect against muscle loss during prolonged periods of inactivity [341]. The use of leucine to supplement meals with insufficient protein content to maximally stimulate MPS may be a useful, cost-effective, and acceptable strategy to maintain muscle mass during lockdown.

#### Creatine

Creatine (Cr) is a non-protein amino acid found in red meat and seafood [342] and it is widely used as an ergogenic aid for athletes [343]. In the body, Cr combines with a phosphoryl group to form phosphocreatine (PCr). Elevated muscle levels of PCr help to maintain ATP availability through recycling of ADP to ATP, a process essential for maintaining energy availability, particularly during maximal effort anaerobic sprint-type exercise [344]. Creatine supplementation has been shown to be particularly beneficial for strength and power athletes [345] and a number of its ergogenic effects may be useful for countering the muscle mass and functional losses associated with sarcopenia/aging, namely:

- Improved performance in sets of high-intensity muscle contractions
- Increased muscle mass and strength adaptations from training
- Enhanced recovery
- Greater training tolerance [346]

Creatine supplementation has been shown to be safe and effective for improving accrual of LM and improving strength in older people [276]. A study by Aguiar et al. [347] in healthy women (mean age 65 years) undergoing a 12-week RE program reported that those supplementing with 5 g of creatine per day experienced a greater improvement in bench press, knee extension, and bicep curl 1RM strength and improvements in functional performance, as well as a greater increase in muscle mass (+ 2.8 kg) than the control group. It has also been observed that plasma and muscle creatine levels are lower in those who eat vegetarian/low-meat diets [348] and older populations [349], thus highlighting the importance of supplementing creatine in these groups. Creatine’s safety and cost-effectiveness make it a potentially useful supplement, to take in conjunction with a (home based) RE protocol, for the prevention of muscle atrophy and sarcopenia [350].

#### Long-chain, omega-3 fatty acids

Eicosapentaenoic acid (EPA) and docosahexaenoic acid (DHA) are omega-3 fatty acids of marine origin that are widely investigated for their potential health benefits in conditions such as CVD, cognitive decline, chronic inflammation, and depression [351–354]. Long-chain, marine, omega-3 fatty acid supplementation has also been shown to augment the MPS response to protein ingestion in younger and older adults [355, 356]. There is evidence that this effect is partially mediated via activation of the mTORc1-S6K1 signaling pathway which is essential in the process of protein synthesis and muscle growth [77, 356]. This indicates that sufficient omega-3 supplementation may, at least partially, be able to counter the anabolic resistance typical of the aging process. Clinical trials have added evidence for this possibility. After a 3-month RE program with 45 healthy women (mean age 64 years), the two groups supplementing with 2 g of fish oil per day experienced greater improvements in muscle strength and functional capacity compared to a control group [357]. Similarly, high-dose fish oil supplementation (4 g per day) has been shown to increase thigh muscle volume and grip strength in older men and women (mean age 68 years) despite no RE protocol being included in the trial [358]. Thus, long-chain, marine, omega-3 fatty acids may be a useful adjunct strategy to overcoming the anabolic resistance-induced losses in muscle mass that are observed in aging. It should be noted however, that a number of the trials mentioned here used a particularly high-grade and high-dose (4 g per day) omega-3 supplement known as Lovaza [355, 356, 358] and, accordingly, such high doses may be necessary to achieve a physiologically significant effect. Due to their anti-inflammatory effects, omega-3 supplementation may offer the further benefit of managing the “cytokine storm” observed in severe COVID-19 infections [359] and has already been suggested as an adjuvant therapy [360]. Furthermore, the use of EPA or EPA/DHA combinations is recommended in the treatment of mood disorders, which may be more common during COVID-19 confinement [361].

#### Vitamin D

The relevance of vitamin for muscle health has already been discussed but we will briefly mention the results of trials investigating the effects of vitamin D supplementation on muscle mass and function. In a 6-month intervention in institutionalized older adults (≥ 60 years) with vitamin D deficiency, those receiving vitamin D improved hip flexor strength by 16.4% and knee extensor strength by 24.6% without any RE protocol [362]. This was in contrast to the control group which received no vitamin D and reported no improvement in strength. The dosage in this trial averaged approximately 3666 IU of oral vitamin D3/day. Similarly, in a 9-month study of an older population (≥ 70 years), vitamin D supplementation (400 IU vitamin D3/day) was reported to improve timed up and go performance and gait speed compared to controls [363]. As older adults may require higher doses of vitamin D3 to achieve adequate serum levels (30 ng/mL) [364] and supplementation is safe up to 10,000 IU/day (upper limit of safety), an intake of 1000–4000 IU/day may be suitable, based on current evidence. As low vitamin D status is a potential risk factor for COVID-19 infection [185], supplementation may be a pragmatic strategy for reducing risk of both sarcopenia and COVID-19.

### Energy balance

In addition to encouraging higher intakes of protein, older individuals may need to reduce total calorie intake in order to avoid excess accumulation of body fat due to the potential reduction in activity levels caused by social distancing and quarantine measures [43, 291]. Reducing total calorie intake through a reduction in portion sizes and snacking occasions may be effective methods for maintaining energy balance in the elderly [365]. Maintaining higher protein intakes may be particularly beneficial for avoiding the loss of lean mass during such calorie restriction [366, 367], especially when combined with (home-based) RE which is known to help preserve LM [368, 369]. Where possible, focusing on more whole foods, such as fruit, vegetables, whole grains, and legumes, has been shown to help reduce ad libitum food intake, while also benefiting cardiometabolic health [370]. Higher protein intakes (lean meats, fish, low-fat dairy, etc.) and higher fiber foods (vegetables, fruit, whole grains, legumes, etc.) can also help reduce feelings of hunger that may arise from reduced caloric intake, improving adherence and helping to avoid body fat gain [338, 371]. Similarly, reducing UPFs may be a useful strategy to reduce excessive consumption of food and weight gain [133].

Telehealth services aimed at promoting improved dietary habits may also be beneficial as the addition of supervision and behavioral support is known to enhance the effectiveness of dietary advice [372, 373]. While there is evidence to suggest that these dietary telehealth strategies are effective, there is also evidence to suggest that certain individuals may find “no-contact” approaches to be more effective [85, 374]. This should be considered when providing older people with appropriate support and guidance, in order to better tailor advice to their needs and circumstances.

## Conclusions

The COVID-19 pandemic has and will continue to have wide-reaching repercussions on all aspects of society. While social distancing and isolation measures implemented by governments are necessary for the greater societal good, governments also have a responsibility to provide some form of care for those that are quarantined or isolated and, in particular, those at greatest risk of infection [375]. Reductions in physical activity, disruption to normal eating habits, stress, and altered sleeping patterns will put older people at greater risk of sarcopenia which, along with its own implications for quality of life and mobility, can lead to the progression of multiple lifestyle-related diseases. Many of those hospitalized by COVID-19 will also suffer from some degree of muscle loss and will likely require some form of rehabilitation to regain that lost muscle mass and function [376]. In this review, we have highlighted some of the primary causes of muscle loss and sarcopenia. Their relevance to both short- and long-term health burden, as well as their relevance to the risk of contracting COVID-19, or experiencing worsened outcomes post-infection, should be recognized and considered carefully by governmental and public health bodies. We have also suggested some of the most useful and practical, evidence-based counter measures that can be safely implemented to reduce the progression of sarcopenia, improve physical function and well-being, and potentially reduce the risk and severity of infection. Physical activity will play a key role and tailoring such programs to the needs and abilities of the participants will be vital. This highlights the importance of online and phone-based virtual care and telehealth services, which have become common place in standard medical care during this pandemic [377]. This digital health framework can be leveraged to provide older adults with the remote supervision and guidance needed to encourage the adoption of the exercise habits and dietary practices necessary for musculoskeletal health. Subsidization or outright provision of such online support services as well as their promotion amongst those that need it most should be considered by governments and local authorities, as should subsidization of low-cost equipment that may improve uptake of said services. The potential for under-, over-, and malnutrition during COVID-19 lockdown is also very real, especially amongst disadvantaged groups, and governments must consider policies to ensure that people have access to sufficient, reasonably priced, high-protein, predominantly whole foods in order to maintain muscle mass and avoid energy imbalances leading to either excess fat accumulation or unnecessary body weight loss. Like many difficult global health problems, the solutions may be apparent but the logistics of implementing them may be lacking. Success in counteracting the risk of muscle loss caused by the pandemic will be determined by our capability to develop efficient strategies that can protect vulnerable populations and maintain or improve the health status of the populace at large.

## Data Availability

Not applicable.
